# Breathable, Flexible, and Durable Woven Fabric with Polyester/Carbon Nanotube-Wrapped Cotton Yarns for Wearable Electrothermal Heaters

**DOI:** 10.3390/polym18192454

**Published:** 2026-10-08

**Authors:** Yunxia Liang, Xuejiao Wang, Qiwen Liu, Ya Wang, Yuexiao Sun, Yiming Xu, Dianliang Wang, Xiaogang Luo, Ke-Qin Zhang

**Affiliations:** 1 National Engineering Laboratory for Modern Silk, College of Textile and Clothing Engineering, Soochow University, 199 Ren-Ai Road, Suzhou 215123, China; 2 PPM Institute of Functional Materials, Poly Plastic Masterbatch (Suzhou) Co., Ltd., 29 Xujiaguan Road, Suzhou 215144, China

**Keywords:** wearable heaters, blended fabric, carbon nanotube-wrapped cotton yarn, electrothermal conversion performance, Joule heat effect

## Abstract

Owing to their breathability, flexibility, washability, and wearability, fabric-based wearable heaters have gained significant attention for personal thermal management and thermotherapy applications. A key challenge, however, lies in achieving high electrical conductivity without compromising the inherent fabric properties or electrothermal performance. To address this, we applied a surface engineering strategy by weaving polyester (PET) warp yarns with carbon nanotube-wrapped cotton weft yarns (CCY) to construct a conductive blended fabric. The CCY was prepared through a facile surface wrapping process, creating a conductive layer on the cotton yarns. The resulting PET/CCY fabric exhibited excellent breathability, with air permeability of 1104.09 ± 7.69 mm s^−1^ and moisture permeability of 2536.67 ± 25.45 g m^−2^ day^−1^. It also demonstrated superior electrothermal performance, including a rapid response time (45 s), low driving voltage (1–7 V), uniform temperature distribution, and reliable stability. Remarkably, the fabric maintained consistent performance after 5000 bending cycles, 500 folding cycles, 60 washing cycles, and 12 weeks of atmospheric exposure, highlighting its flexibility, washability, and long-term durability. These comprehensive properties position the PET/CCY blended fabric as a promising high-performance wearable heater for advanced personal thermal management and thermotherapy applications.

## 1. Introduction

In recent years, wearable heaters based on the Joule heat effect have gathered significant attention from both academia and industry due to their promising applications in diverse fields such as personal thermal management, wearable thermotherapy, visual indication and thermal camouflage [1,2,3,4,5]. Among these, fabric-based wearable heaters are of special interest owing to their breathability, flexibility, washability and ease of integration, enabling advanced wearability in practical applications [6,7,8,9]. However, common textile fabrics like cotton and polyester (PET) fabrics are usually electron insulating and phonon insensitive, which make them unsuitable for use as wearable heaters [10,11]. In this regard, the key to constructing fabric-based wearable heaters lies in endowing such textile fabrics with high conductivity while retaining their inherent properties, including breathability, flexibility, and washability [12,13].

Currently, there are two main strategies for the fabrication of conductive textile fabrics for wearable heaters. The first strategy involves compositing commercially available textile fabrics with various conductive materials, including metal nanowires/nanoparticles [14,15], carbon nanotube (CNT)/graphene-based nanomaterials [16,17,18,19], conductive polymers [20,21], MXene [10,22,23] and their hybrids [24,25,26], through different methods. For example, wang et al. [10] developed a conductive fabric by dip-coating MXene onto a PET fabric, and the resulting fabric exhibited a good electrothermal conversion performance including a wide temperature range (40–174 °C), low driving voltage (1–3.5 V), and fast response time (<25 s). Although this strategy offers advantages in terms of fabrication simplicity and rapid conductivity enhancement, there is a potential risk of the conductive materials detaching during the repeated mechanical deformations and washing due to the poor interfacial bonding between the fabric matrix and the conductive materials, which will greatly limit the long-term durability of the fabrics [6]. Meanwhile, the introduced conductive materials could inevitably impair the breathability and flexibility of the fabrics [27,28]. The second strategy involves weaving or knitting polymeric yarns with conductive fibers/yarns into a blended fabric [29,30,31]. For instance, Qu et al. [30] developed a conductive blended fabric by weaving cotton warp yarn and PEDOT: PSS@MXene@cotton weft yarn, and the obtained fabric showed a stable electrothermal conversion performance with a wide temperature range (31–196.8 °C) and low driving voltage (0.5–2.5 V). Although the conductive blended fabrics show good breathability and flexibility, the breakage of the conductive fibers/yarns during the weaving/knitting process is a major concern [28]. Moreover, the electrothermal conversion performance and washability of the blended fabrics are still unsatisfactory [32,33]. Consequently, it remains a great challenge to develop a conductive textile fabric that could simultaneously achieve good electrothermal conversion performance, breathability, flexibility, washability and long-term durability for wearable heaters.

In our previous work, we reported CNT-wrapped polymeric yarns, which was made by wrapping polymeric yarns with a CNT cylinder that was continuously prepared using a floating catalyst chemical vapor deposition (FCCVD) method [34]. This wrapping process ensures that the layers of CNTs can be firmly attached to the polymeric yarns even after knitting. Inspired by the excellent mechanical properties of CNT-wrapped polymeric yarns, here, we designed and fabricated a conductive blended fabric by weaving PET yarns and CNT-wrapped cotton yarns (CCY) using a rapier loom for wearable heaters. Benefiting from the good conductivity of the CCY and the porous woven structure, the resulting PET/CCY blended fabric exhibited not only good breathability of 1104.09 ± 7.69 mm s^−1^ (air) and 2536.67 ± 25.45 g m^−2^ day^−1^ (moisture), but also an excellent electrothermal conversion performance including fast electrothermal response, low driving voltage, uniform temperature distribution as well as heating reliability and stability. Moreover, it could maintain a stable electrothermal conversion performance even after 5000 bending cycles, 500 folding cycles, 60 washing cycles, and 12 weeks of continuous exposure to atmospheric conditions, showing good flexibility, washability and long-term durability. The application capabilities of these fabric-based wearable heaters were demonstrated by sewing them into garments for personal thermal management and wearable thermotherapy.

## 2. Experimental Section

### 2.1. Preparation of CCY and PET/CCY Blended Fabric

The CCY was prepared following our previous work [34]. In brief, a continuous CNT cylinder was firstly produced via an FCCVD method. Subsequently, a cotton yarn was used as a substrate wire to collect the cylinder. When the cylinder came into contact with the ethanol-prewetted substrate wire, it condensed immediately due to the strong contraction force induced by ethanol, and this condensation led to the wrapping of a layer of CNTs onto the substrate wire. To wrap more layers of CNTs, the previously wrapped wire was used as the substrate wire, and the condensation process was performed again. After wrapping five layers of CNTs, the resulting CCY was utilized as the weft yarn, and woven with PET warp yarn to fabricate a PET/CCY blended fabric using a rapier loom. The sources, purities, concentrations, and CAS numbers of all materials are summarized in Table S1. The linear densities, twist directions/levels, and single/ply specifications of the cotton and PET yarns are summarized in Table S2.

### 2.2. Characterization and Tests

The morphology was observed using a scanning electron microscope (SEM, Regulus 8100, Hitachi, Tokyo, Japan). Prior to SEM observation, the yarn or fabric samples were fixed on an electron microscope sample stage with conductive carbon tape and sputter-coated with Au using an ion sputter (MC1000, Hitachi, Tokyo, Japan) at 10 mA for 15 s. The resistance was measured at room temperature via a Keithley 7510 multimeter (Tektronix, Cleveland, Ohio, USA). The air permeability tests were performed on a fully automatic permeability instrument (YG461G, Ningbo Textile Instrument Factory, Ningbo, China) under the Chinese standard GB/T 5453-1997 [35]. The moisture permeability measurements were conducted on a fabric moisture permeability meter (YG(B)216T, Wenzhou Jigao Testing Instrument Co., Ltd., Wenzhou, China) under the Chinese standard GB/T 12704.2-2009 [36]. For the electrothermal conversion performance test, both ends of the sample (4 × 1 cm^2^) were encapsulated with copper foil. A constant voltage was applied to the sample by a DC power supply (IT6832, ITECH Electronic Co., Ltd., Nanjing, China), and the sample temperature was monitored using an infrared thermal imager (Fotric 226, Shanghai Thermal Imaging Technology Co., Ltd., Shanghai, China). For the flexibility test, the sample was subjected to bending and folding deformations, while its electrothermal conversion performance was simultaneously measured. The tensile test was conducted on a single fiber tensile tester (XT08, Shanghai Xuesai Instrument Co., Ltd., Shanghai, China), and the test gap is 20 mm and the stretching rate was 20 mm min^−1^.

For the washability test, the sample was placed in a stainless-steel container with 200 mL of water, 50 steel balls and detergent. The container was loaded into a fastness to washing tester (SW-24, Ningbo Textile Instrument Factory) and washed at 40 °C for 30 min. After washing, the sample was dried in an oven at 50 °C for 180 min. The electrothermal conversion performance of the sample was recorded every 5 washing/drying cycles (i.e., after 0, 5, 10, 15, …, 60 cycles). It should be noted that these 60 cycles refer to 60 accelerated washing/drying cycles adapted from AATCC TM 61-1A [33], and they should not be regarded as equivalent to 60 conventional household washes unless a specific calibration is provided. For the durability test, the sample was exposed directly to atmospheric conditions with the electrothermal conversion performance recorded weekly during the exposure period.

## 3. Results and Discussion

### 3.1. Preparation and Characterization of PET/CCY Blended Fabric

**Figure 1:**
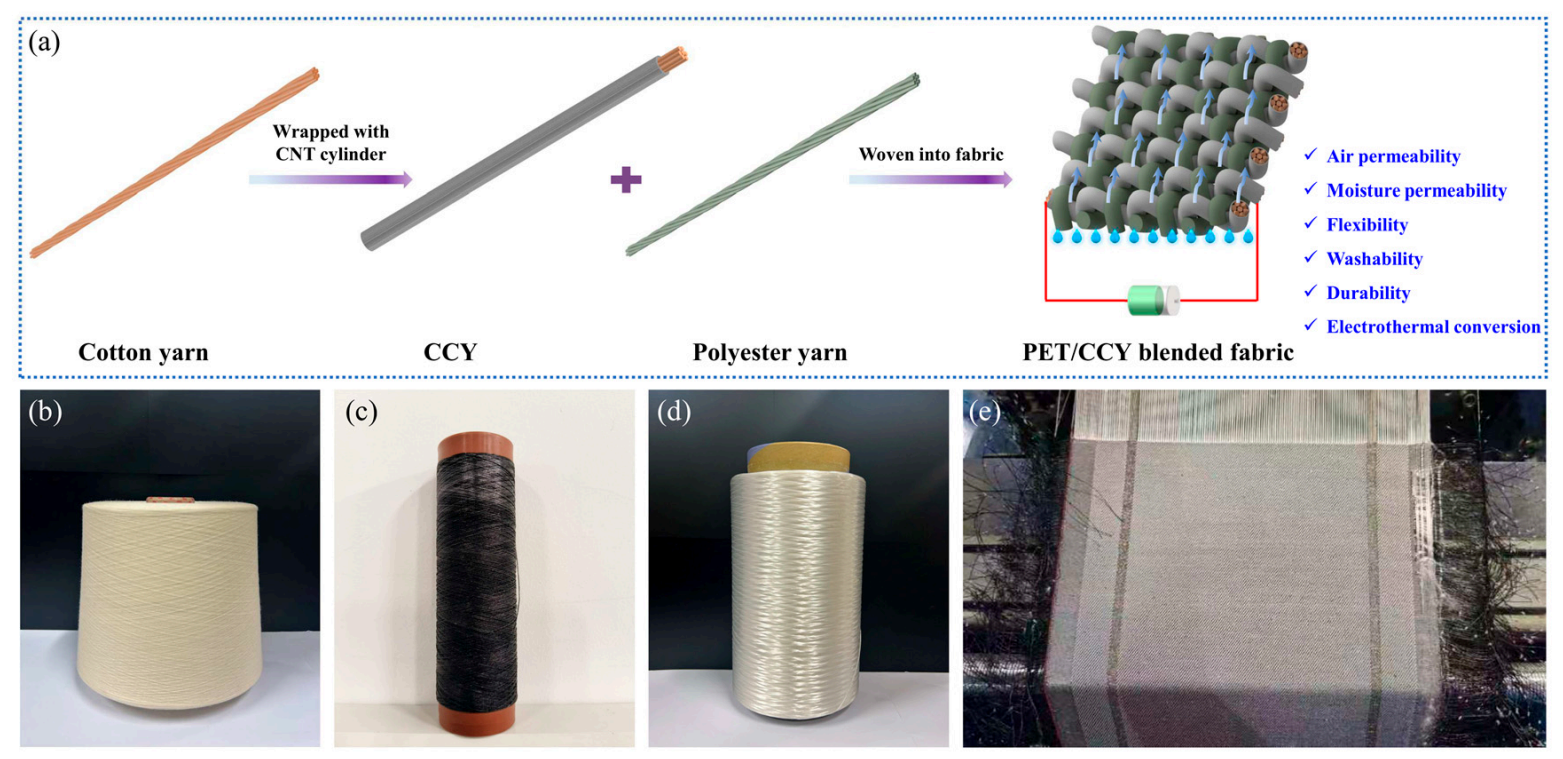
(**a**) Schematic diagram of the preparation process of PET/CCY blended fabric. Optical images of (**b**) cotton yarn, (**c**) CCY, and (**d**) PET yarn. (**e**) Photograph showing the weaving process of the PET/CCY blended fabric.

The preparation process of the PET/CCY blended fabric is schematically illustrated in Figure 1a. Initially, a continuous CNT cylinder was fabricated using a FCCVD technique [37,38]. Then, cotton yarn (Figure 1b) was used as the substrate wire to collect the cylinder for the manufacture of CCY. Upon contact with the ethanol-prewetted cotton yarn, the cylinder condensed immediately because of the strong contraction force induced by ethanol, and this condensation resulted in the wrapping of a layer of CNTs onto the cotton yarn (more details can be found in our previous work [34]). To wrap more layers of CNTs, the previously wrapped cotton yarn was used as the new substrate wire, and the condensation process was conducted again. Figure 1c and Figure S1 present the optical and SEM images of CCY wrapped with five layers of CNTs. These images clearly demonstrate that CNTs were uniformly wrapped over the cotton yarn (Figure S1a,b), yielding a well-defined core–shell structure (Figure S1c). Moreover, owing to the good conductivity of the constituent CNTs, the resulting CCY was highly conductive with a linear resistance of 20.68 Ω cm^−1^ (Figure S1d). Finally, the obtained CCY was employed as the weft yarn and woven together with the PET warp yarn (Figure 1d) to produce a conductive PET/CCY blended fabric using a rapier loom (Figure 1e and Video S1). We therefore expected that the resultant PET/CCY blended fabric would exhibit outstanding air permeability, moisture permeability, flexibility, washability, durability, and electrothermal conversion performance.

**Figure 2:**
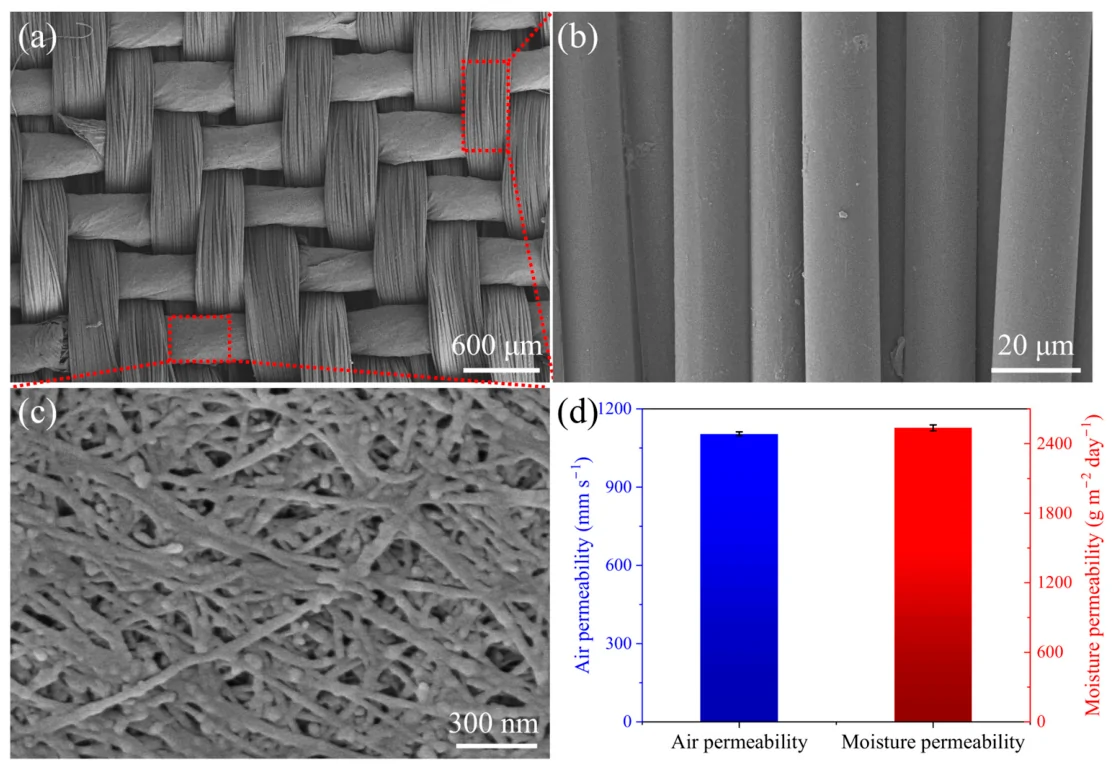
(**a**–**c**) SEM images of the PET/CCY blended fabric. SEM images were acquired at an accelerating voltage of 5.0 kV and a working distance of 10–11 mm. (**d**) The air permeability and moisture permeability of the PET/CCY blended fabric.

The surface morphological features of the PET/CCY blended fabric were studied by SEM. As illustrated in Figure 2a, the blended fabric exhibited a 2/2 twill weave pattern, wherein the PET warp yarn (Figure 2b) and the CCY weft yarn (Figure 2c and Figure S2) interlaced, forming a porous structure. The diameter distribution of CNT bundles on the surface of CCY was measured using ImageJ 1.54p (National Institutes of Health, Bethesda, MD, USA) (Figure S3). A total of 100 CNT bundles were randomly selected from multiple regions of Figure 2c. The mean bundle diameter is 36.19 ± 10.19 nm, indicating a relatively uniform CNT network on the yarn surface. Furthermore, it is noteworthy that the CNT layer remained firmly attached to the cotton yarn surface, even in the presence of noticeable structural deformation in the CCY resulting from stresses incurred during the weaving process. This phenomenon demonstrates a strong binding between the CNT layer and the underlying cotton yarn substrate. Considering that air permeability and moisture permeability are critical factors determining the wearing comfort of textiles, systematic and repeatable measurements of these two parameters were performed on the blended fabric, with the corresponding results presented in Figure 2d. The air permeability of the PET/CCY blended fabric was 1104.09 ± 7.69 mm s^−1^, which surpasses that of previously reported fabric-based wearable heaters, such as graphene/CNT-coated cotton fabric (331.35 mm s^−1^) [17], MXene@PU/PET blend fabric (295.87 mm s^−1^) [39], and sericin-MXene-coated silk fabric (315.16 mm s^−1^) [40]. The moisture permeability of the PET/CCY blended fabric was measured to be 2536.67 ± 25.45 g m^−2^ day^−1^, which satisfies the requirement concerning moisture permeability for wearable fabrics (≥2500–5000 g m^−2^ day^−1^) [41]. These findings collectively confirm that the PET/CCY blended fabric we developed demonstrates outstanding breathability, thereby ensuring sufficient comfort when worn on various regions of the human body.

### 3.2. Electrothermal Conversion Performance of PET/CCY Blended Fabric

**Figure 3:**
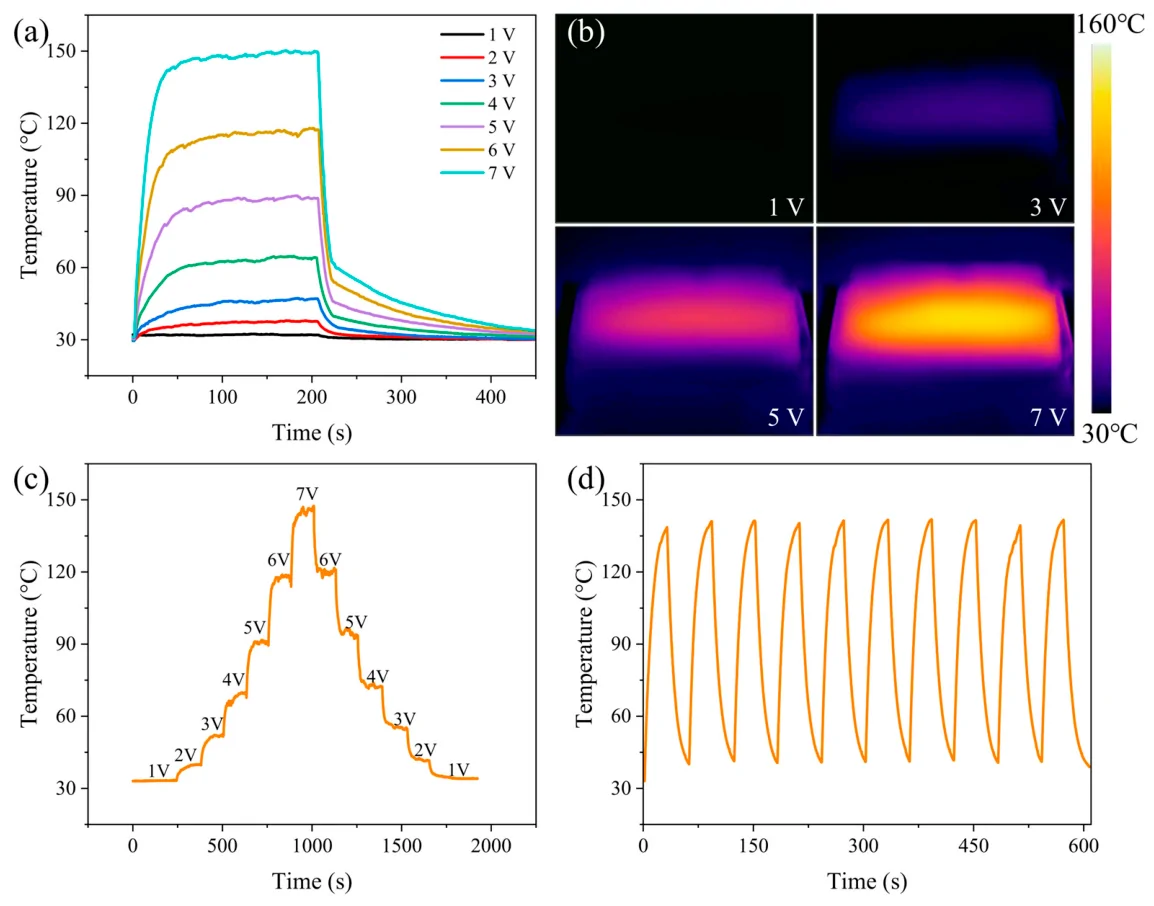
Electrothermal conversion performance of the PET/CCY blended fabric (4 × 1 cm^2^). (**a**) Temperature–time curves at different applied voltages. (**b**) The corresponding infrared images at the voltages of 1 V, 3 V, 5 V, and 7 V. (**c**) Temperature response to stepwise voltage variation. (**d**) Temperature response during 10 cycles of repeated heating and cooling at a voltage of 7 V.

By virtue of the good conductivity of the CCY, we investigated the electrothermal conversion performance of the PET/CCY blended fabric (4 × 1 cm^2^) under atmospheric conditions for potential application in wearable heaters. Figure 3a shows the temperature–time curves of the blended fabric at different applied voltages. As can be observed, the blended fabric generated heat immediately when a voltage was supplied and reached the saturation temperature within 45 s, demonstrating its rapid electrothermal response. The saturation temperature increased with the increasing applied voltages, since more Joule heating power was generated according to Joule’s law (*P* = *U*^2^/*R*, where *P* is the Joule heating power, *U* is the applied voltage, and *R* is the resistance) [4,15], and exhibited an excellent linear relationship with the square of the applied voltage (Figure S4). In particular, the saturation temperature, which is suitable for the human body (core 37 °C and skin 33 °C) [42], can be achieved at a very low driving voltage of 1 V (33 °C) and 2 V (40 °C), respectively. Such a low voltage not only ensures the safety of the human body, but also makes it possible for the blended fabric to be driven by a wearable energy supply device [43]. At a supplied voltage of 7 V, the saturation temperature reached a maximum of 147 °C. With a rapid response time (45 s) and a high saturation temperature (147 °C), the PET/CCY blended fabric outperforms some reported CNT-based electrothermal fabrics, such as PTFE/CNT fiber woven textile (>500 s, 145 °C) [44], Cotton@PDA/CNTs/PFOTES (<175 s, >85 °C) [18], CNT/cellulose/CuNW/cotton fabric (80 s, 70 °C) [45], and CNT@silk fabric (<125 s, 42.8 °C) [16] (Table S3). Meanwhile, the corresponding infrared images in Figure 3b and Figure S5 revealed that the temperature distribution across the blended fabric was uniform, which can be ascribed to the uniform wrapping of CNTs over the cotton yarn. Figure 3c,d illustrates the temperature response of the blended fabric when subjected to stepwise-changed voltages and 10 cycles of repeated heating and cooling at 7 V, respectively. The reproducible temperature indicated that the blended fabric had an excellent heating reliability, repeatability and stability. All the above results clearly verify that our PET/CCY blended fabric possesses an excellent electrothermal conversion performance, including fast electrothermal response, low driving voltage, uniform temperature distribution as well as heating reliability and stability, and thus holds great promise for use as wearable heaters.

### 3.3. Flexibility, Washability and Durability of PET/CCY Blended Fabric

**Figure 4:**
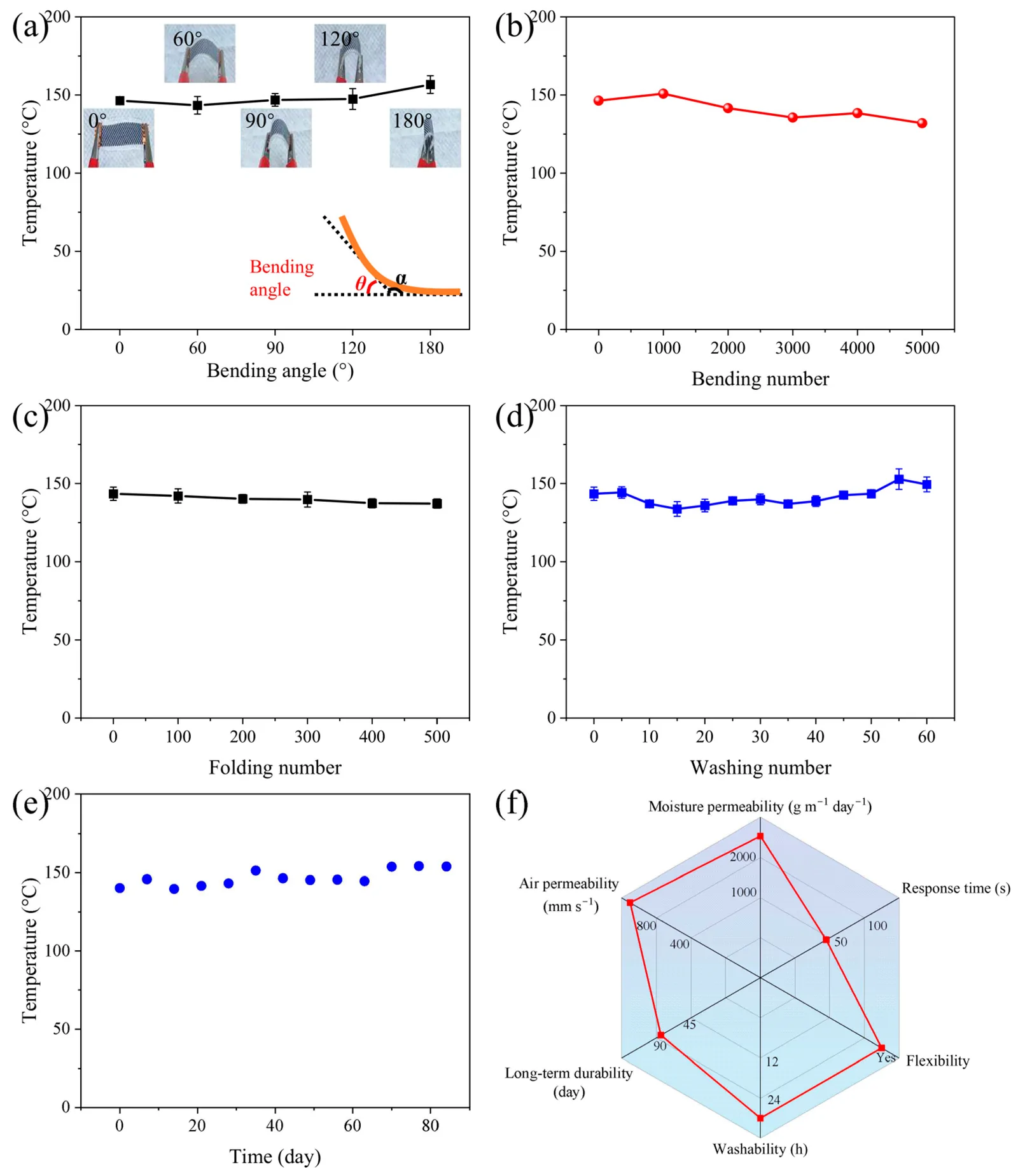
(**a**) The temperature changes in the blended fabric at discrete bending angles of 0°, 60°, 90°, 120°, and 180°. The inset shows a schematic diagram and photographs of the blended fabric at the corresponding bending angles. The temperature evolution of the blended fabric during (**b**) 5000 bending cycles, (**c**) 500 folding cycles, (**d**) 60 washing cycles, and (**e**) 12 weeks of continuous exposure to atmospheric conditions at a supplied voltage of 7 V. (**f**) The performance of our PET/CCY blended fabric-based wearable heater.

In addition to the basic electrothermal conversion performance, the flexibility, washability and long-term durability also need to be considered for the practical application of a wearable heater [10,46]. To evaluate the flexibility of the PET/CCY blended fabric, it was subjected to both bending and folding deformations, while the electrothermal conversion performance was measured simultaneously. Figure 4a records the temperature changes in the blended fabric as a function of the bending angle at a supplied voltage of 7 V. The bending angle *θ* is defined as the change in the tangent direction of the sample relative to its flat state (*θ* = 180° − *α*, where *α* is the internal angle between the two arms of the sample). Therefore, *θ* = 0° represents the flat state and *θ* = 180° represents the fully folded state. Temperature measurements were performed at discrete bending angles of 0°, 60°, 90°, 120°, and 180°. Apparently, as the bending angle increased, the temperature remained almost unchanged within the experimental uncertainty. The infrared images shown in Figure S6 also revealed that there was no obvious change in the temperature and the temperature distribution under different bending angles. Even after 5000 cycles of bending at 90°, the blended fabric could retain 90% of its initial temperature (Figure 4b). Additionally, as depicted in Figure 4c, the blended fabric also could maintain 95% of its initial temperature value even after folding for 500 cycles, without any obvious changes in the structural integrity and morphology (Figure S7). All of these results suggested that the blended fabric exhibited excellent mechanical flexibility. To further understand the mechanical origin of the fabric flexibility, tensile tests were also performed on pristine cotton yarn and CCY (Figure S8). Pristine cotton yarn had a tensile strength of 79.81 ± 1.15 MPa and an elongation at break of ~9.8%, while CCY reached 174.61 ± 6.89 MPa but decreased to ~7.7% elongation. The higher initial modulus and sharp fracture of CCY indicate increased stiffness and reduced ductility. Although the CNT coating reduced the ductility of the cotton yarn to some extent, the woven PET/CCY fabric still maintained good flexibility, as confirmed by the bending and folding tests.

To assess the washability of the PET/CCY blended fabric, it was subjected to washing according to the AATCC test standard TM 61-1A Test Method for Colorfastness to Laundering: Accelerated [33]. Specifically, 50 steel balls were added to simulate extreme washing conditions. The electrothermal conversion performance was then tracked for 60 washing cycles. As shown in Figure 4d, the temperature remained almost constant within the margin of error of the experiment, indicating the excellent washability of the blended fabric. Moreover, the SEM images illustrated in Figure S9 revealed that the structural integrity and morphology of the blended fabric were still well maintained even after 60 washing cycles, further highlighting the excellent washability. Such excellent washability of the PET/CCY blended fabric could be attributed to the strong binding between the CNT layer and the underlying cotton yarn substrate.

The long-term durability of the PET/CCY blended fabric was evaluated by exposing it directly to atmospheric conditions with the electrothermal conversion performance monitored weekly. As shown in Figure 4e, over the test period of 12 weeks, the temperature exhibited a slight change within the margin of error of the experiment. In addition, the SEM images revealed no visible changes in the structural integrity and morphology of the blended fabric (Figure S10). These results persuasively demonstrated good long-term durability of the blended fabric in atmospheric conditions. Consequently, the PET/CCY blended fabric exhibits not only exceptional long-term durability but also maintains stable electrothermal conversion performance even after repeated bending, folding and washing, reinforcing its potential for practical wearable heater application. The performance comparison between our PET/CCY blended fabric-based wearable heater and the previously reported fabric-based wearable heaters is listed in Table S3. Importantly, this work does not claim to be the best in every individual metric, but rather provides a balanced combination of electrothermal conversion performance, breathability, flexibility, washability, and long-term durability. To the best of our knowledge, this is among the first reports of a fabric-based wearable heater that simultaneously achieves these properties (Figure 4f).

### 3.4. Applications of PET/CCY Blended Fabric-Based Wearable Heaters

**Figure 5:**
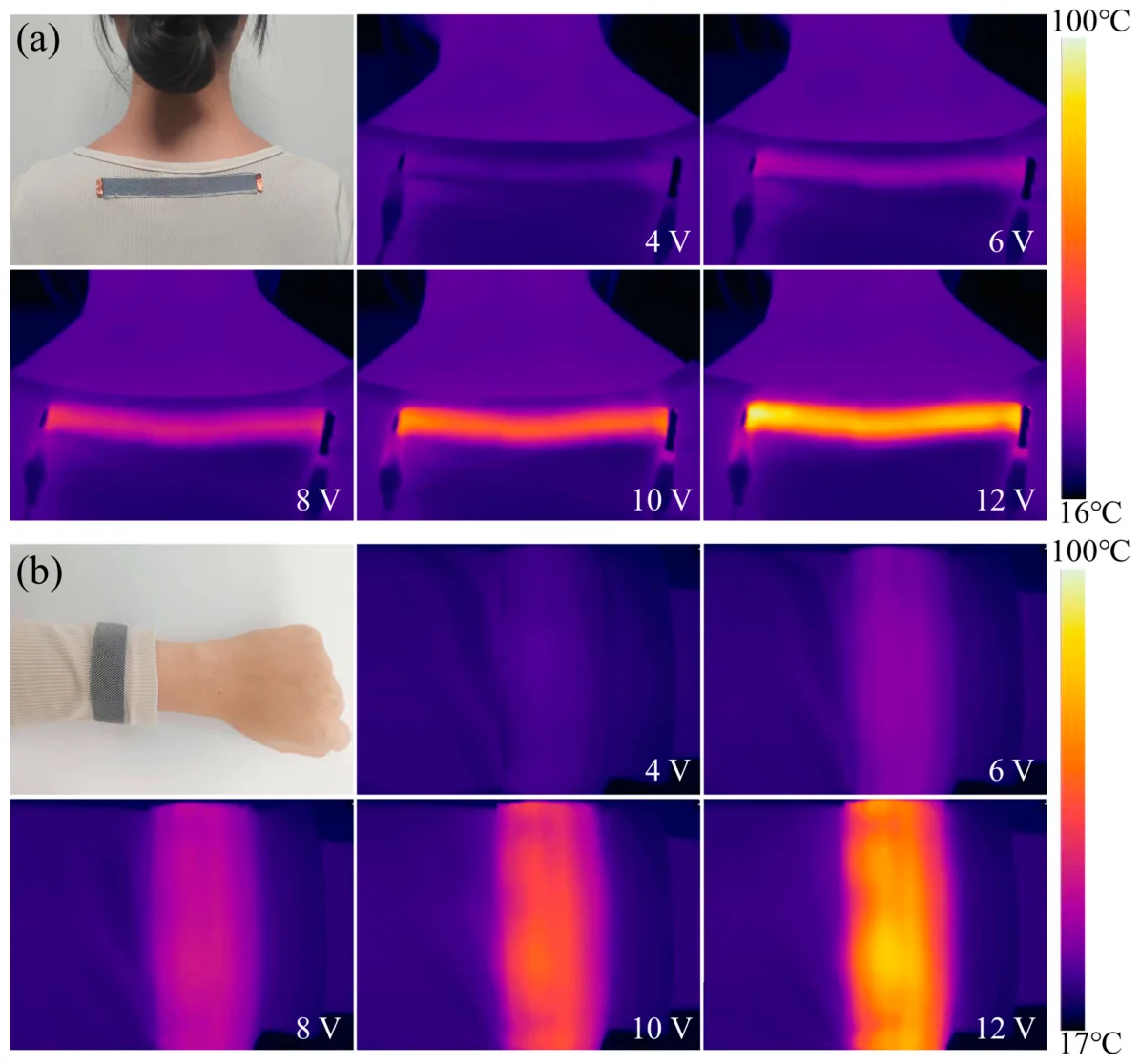
(**a**) Optical image of a blended fabric (15 × 1.5 cm^2^) sewn onto the back region of a garment for personal thermal management and the corresponding infrared images under different voltages. (**b**) Optical image of a blended fabric (15 × 2 cm^2^) sewn onto the arm region of a garment for wearable thermotherapy and the corresponding infrared images at different voltages. The temperatures are surface temperatures of the heater measured under indirect contact conditions (through a fabric layer).

As mentioned above, our PET/CCY blended fabric exhibits excellent comprehensive properties and thus can work as a high-performance wearable heater for personal thermal management and wearable thermotherapy [4,43]. In order to demonstrate these two applications, the blended fabrics of different sizes were integrated into different parts of a garment through simple sewing and then supplied with various voltages. Figure 5a shows the optical image of a blended fabric (15 × 1.5 cm^2^) sewn onto the back region of a garment for personal thermal management and the corresponding infrared images under different voltages. When it was subjected to a voltage of 4 V, the temperature rapidly increased from ambient temperature to 34 °C. This temperature lies in the comfortable temperature range for the human body (33–37 °C) [42] and is therefore suitable for keeping the human body warm in cold environments. Figure 5b shows the optical image of a blended fabric (15 × 2 cm^2^) sewn onto the arm region of a garment for wearable thermotherapy, alongside the corresponding infrared images taken at different voltages. At supplied voltages of 8 and 10 V, the temperature rapidly increased from room temperature to 49 and 65 °C, respectively. These two temperatures are suitable for use in thermotherapy, which usually requires temperatures ranging from 41 to 77 °C to relieve pain and stiffness [47]. To ensure safety, the 65 °C condition was applied only for a very brief duration (<10 s) as a proof-of-concept for rapid heating, while long-term thermotherapy should be conducted below 45 °C. In addition, all the infrared images showed a uniform temperature distribution, which is in good agreement with previous findings. The results indicate that the wearable heaters in this study have great potential for personal thermal management and wearable thermotherapy applications.

## 4. Conclusions

In summary, a conductive blended fabric for wearable heaters was successfully fabricated by weaving PET warp yarns with carbon nanotube-wrapped cotton (CCY) weft yarns. Due to the porous woven structure, the resulting fabric exhibited high breathability, with an air permeability of 1104.09 ± 7.69 mm s^−1^ and a moisture permeability of 2536.67 ± 25.45 g m^−2^ day^−1^. It also demonstrated outstanding electrothermal performance, enabled by the excellent conductivity of the CCY, including rapid response, low driving voltage, uniform heating, and reliable operation. Moreover, the fabric maintained stable electrothermal functionality after extensive mechanical testing (5000 bending and 500 folding cycles), 60 washing cycles, and 12 weeks of atmospheric exposure, confirming its excellent flexibility, washability, and long-term durability. These properties make the PET/CCY fabric a highly promising candidate for wearable heaters in personal thermal management and thermotherapy applications. This work presents a novel fabric design that achieves high electrothermal performance without compromising the inherent textile qualities essential for comfortable wear.

Notably, the developed fabric offers potential sustainability benefits but faces recyclability challenges. Cotton is biodegradable and PET is recyclable, but their blending hinders end-of-life separation. Directly wrapping FCCVD-grown CNTs onto cotton yarn enables the fabrication of conductive yarns, avoiding conductive inks, pastes, or other modifiers and the associated solvent-based coating steps. However, the FCCVD process itself still requires further life-cycle assessment because of its energy consumption, catalyst use, and potential CNT release. For industrial scale-up, limited production throughput, batch-to-batch property variation, and high production cost remain key challenges requiring further study.

Looking forward, future work should focus on reducing energy use and improving process control. Low-temperature or plasma-assisted FCCVD may lower energy demand and broaden yarn compatibility, while green catalysts and renewable carbon sources could reduce environmental impact. Real-time monitoring could improve batch-to-batch consistency. Recyclable textile designs and safe CNT handling should also be developed. Finally, this method could be extended to other natural and synthetic yarns for smart textiles, wearable electronics, and thermal management.

## Data Availability

The original contributions presented in this study are included in the article/Supplementary Materials. Further inquiries can be directed to the corresponding authors.

## Supplementary Materials

The following supporting information can be downloaded at: https://www.mdpi.com/article/10.3390/polym18192454/s1, Figure S1: SEM images of surface and cross-section of the CCY, photograph of an LED being lighted with the CCY as an electric wire; Figure S2: SEM image of the PET/CCY blended fabric; Figure S3: Diameter distribution histogram of CNT bundles on the surface of CCY, measured from SEM images using ImageJ (N = 100); Figure S4: Experimental data and linear fitting of the saturation temperature versus the square of the applied voltage; Figure S5: The infrared images of the PET/CCY blended fabric at the voltages of 2 V, 4 V, and 6 V; Figure S6: The infrared images of the PET/CCY blended fabric at various bending angles; Figure S7: SEM image of the PET/CCY blended fabric after folding for 500 cycles; Figure S8: Mechanical properties of pristine cotton yarn and CCY; Figure S9: SEM images of the PET/CCY blended fabric after washing for 20, 40, and 60 cycles; Figure S10: SEM images of the PET/CCY blended fabric after 12 weeks of continuous exposure to atmospheric conditions; Table S1: Detailed information on all materials; Table S2: Yarn specifications; Table S3: Performance comparison of our PET/CCY blended fabric-based wearable heater with previously reported fabric-based wearable heaters; Video S1: Automatically weaving the CCY weft yarn and PET warp yarn into a conductive blended fabric.
