# Supplementary material for: Breathable, Flexible, and Durable Woven Fabric with Polyester/Carbon Nanotube-Wrapped Cotton Yarns for Wearable Electrothermal Heaters
Source: Polymers (Basel). 2026 Oct 8;18(19):2454. doi: 10.3390/polym18192454 (PMC13667458; doi:10.3390/polym18192454)
Supplement: Supplementary file 1 [file polymers-18-02454-s001.zip › Supplementary Materials.pdf]

Supporting Information

Breathable, Flexible, and Durable Woven Fabric with Polyester/Carbon Nanotube-Wrapped Cotton Yarns for Wearable Electrothermal Heaters

Yunxia Liang[1], Xuejiao Wang[1], Qiwen Liu[1], Ya Wang[1], Yuexiao Sun[1], Yiming Xu[2], Dianliang Wang[2], Xiaogang Luo[1,*], Ke-Qin Zhang[1,*]

[1] National Engineering Laboratory for Modern Silk, College of Textile and Clothing Engineering, Soochow University, 199 Ren-Ai Road, Suzhou 215123, China

[2] PPM Institute of Functional Materials, Poly Plastic Masterbatch (Suzhou) Co., Ltd., 29 Xujiaguan Road, Suzhou 215144, China

* Corresponding author: xgluo@suda.edu.cn (X.L.), kqzhang@suda.edu.cn (K.Z.).

Video S1: Automatically weaving the CCY weft yarn and PET warp yarn into a conductive blended fabric.

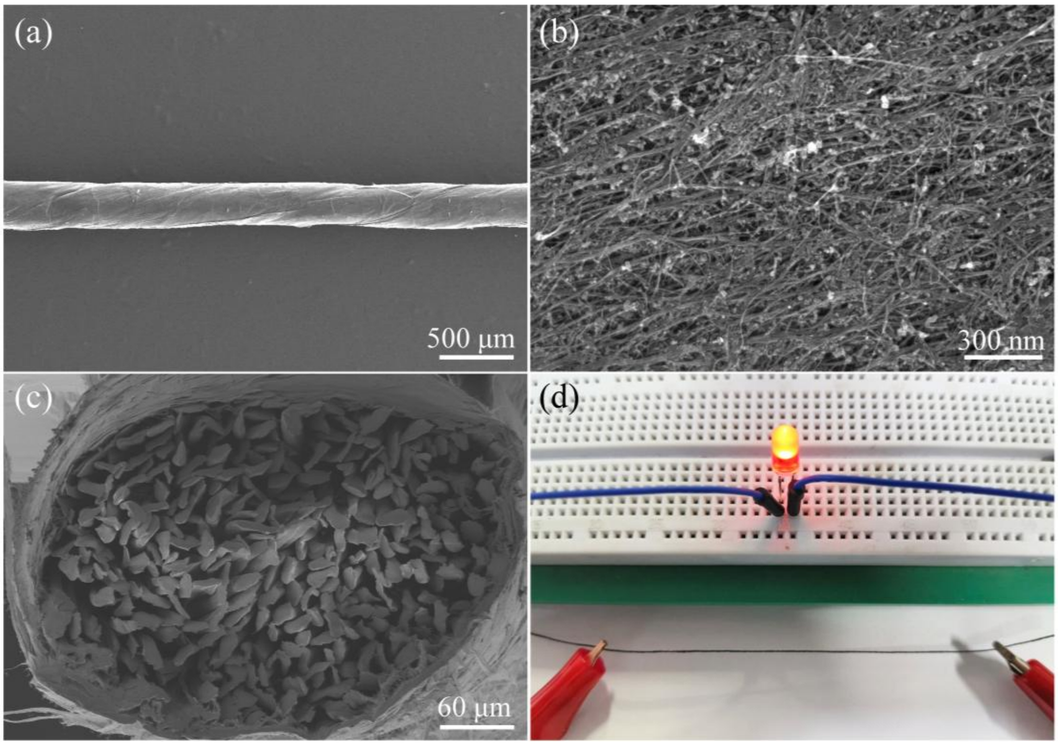

Figure S1. SEM images of (a, b) surface and (c) cross-section of the CCY. SEM images were acquired at an accelerating voltage of 5.0 kV and a working distance of 2 mm. (d) Photograph of a LED being lighted with the CCY as electric wire.

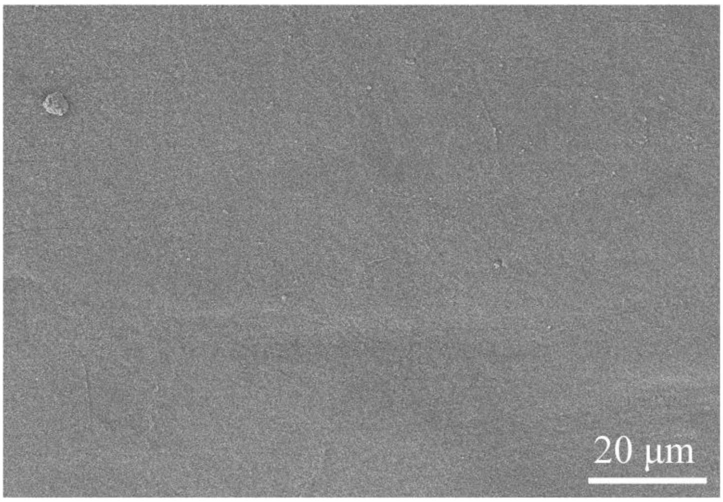

Figure S2. SEM image of the PET/CCY blended fabric. SEM images were acquired at an accelerating voltage of 5.0 kV and a working distance of 10-11 mm.

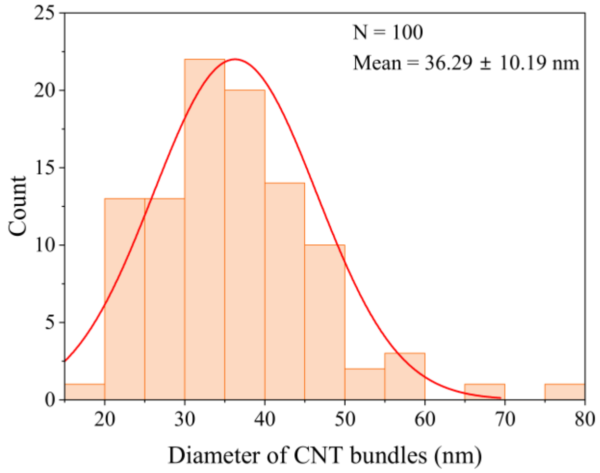

Figure S3. Diameter distribution histogram of CNT bundles on the surface of CCY, measured from SEM images using ImageJ (N = 100). The solid red line represents the Gaussian fitting curve.

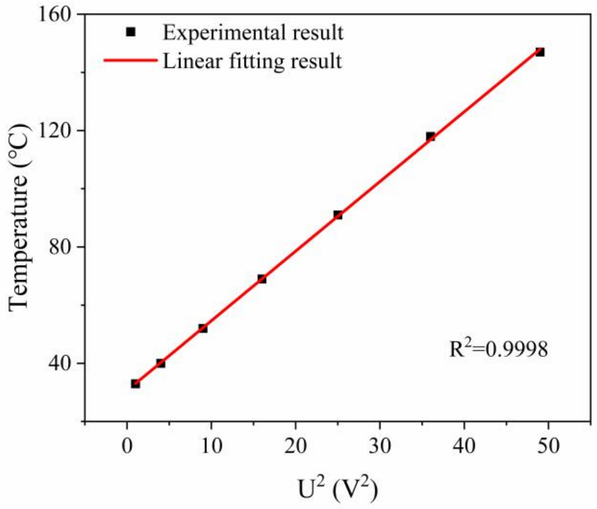

Figure S4. Experimental data and linear fitting of the saturation temperature *versus* the square of the applied voltage (U^2).

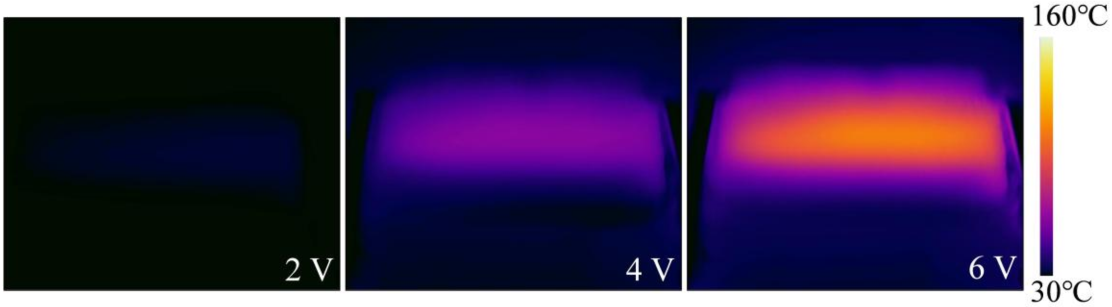

Figure S5. The infrared images of the PET/CCY blended fabric at the voltages of 2 V, 4 V, and 6 V.

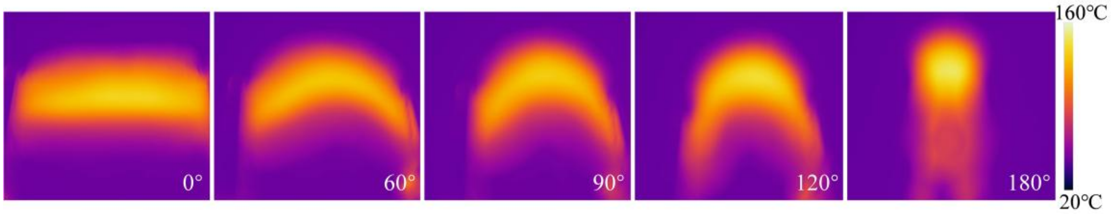

Figure S6. The infrared images of the PET/CCY blended fabric at various bending angles.

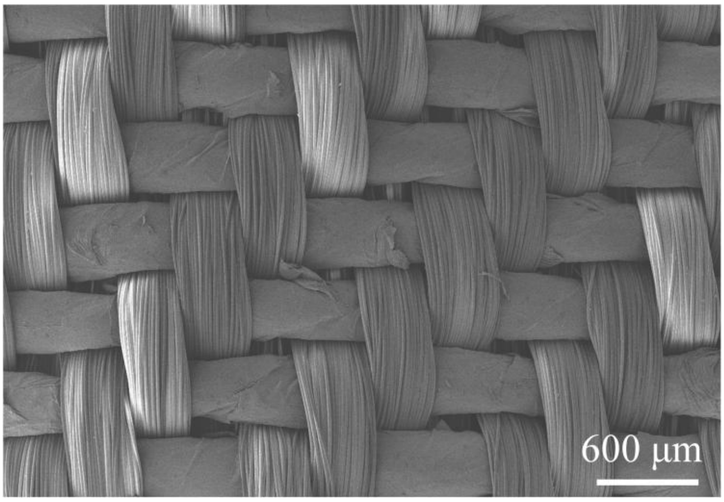

Figure S7. SEM image of the PET/CCY blended fabric after folding for 500 cycles. SEM images were acquired at an accelerating voltage of 5.0 kV and a working distance of 10-11 mm.

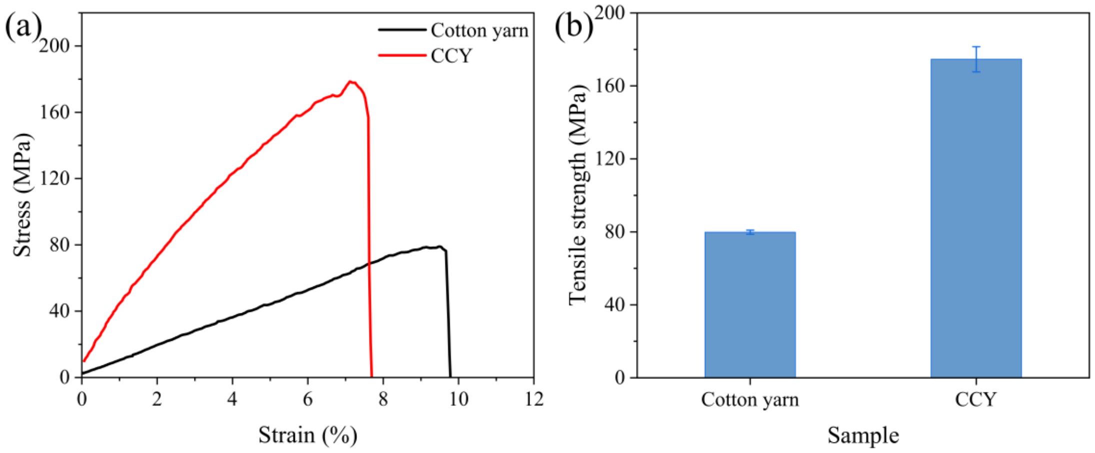

Figure S8. Mechanical properties of pristine cotton yarn and CCY. (a) Typical stress-strain curves of pristine cotton yarn and CCY. For each group, the representative curve was selected from five specimens based on the smallest deviation in tensile strength and elongation at break from the group mean. (b) The average tensile strength of pristine cotton yarn and CCY.

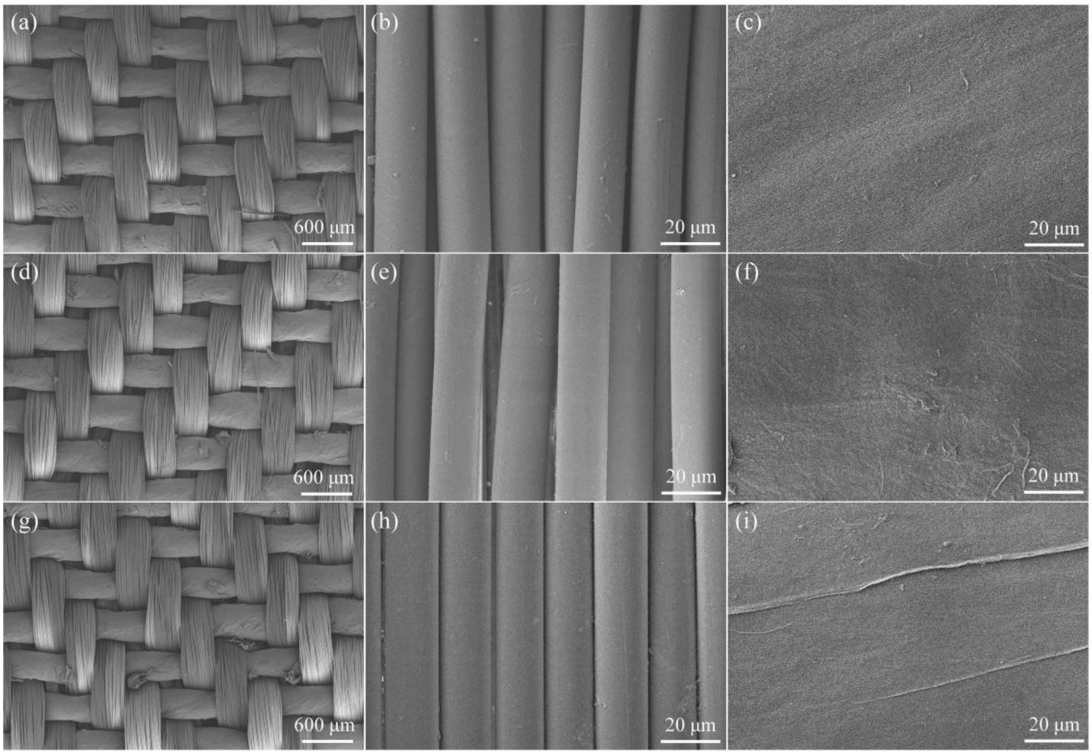

Figure S9. SEM images of the PET/CCY blended fabric after washing for (a-c) 20, (d-f) 40, and (g-i) 60 cycles. SEM images were acquired at an accelerating voltage of 5.0 kV and a working distance of 10-11 mm.

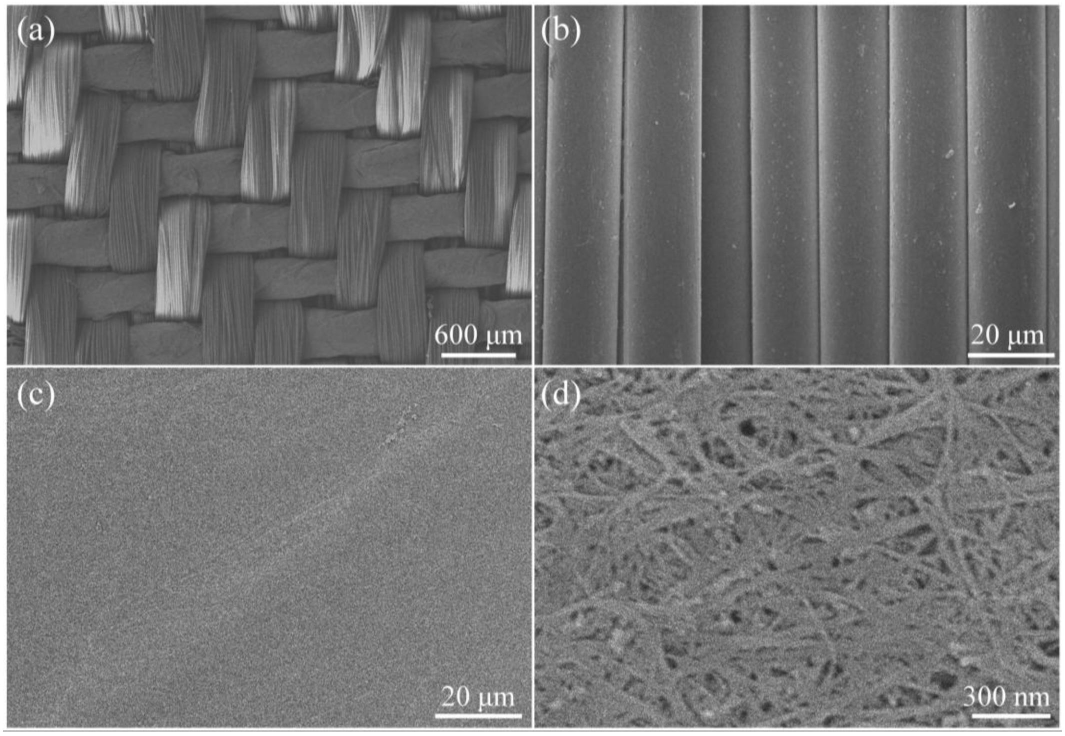

Figure S10. SEM images of the PET/CCY blended fabric after 12 weeks of continuous exposure to the atmospheric conditions. SEM images were acquired at an accelerating voltage of 5.0 kV and a working distance of 9 mm.

Table S1. Detailed information of all materials.

Material	Source/Supplier	Purity	Concentration	CAS number
Ethanol	Sinopharm Chemical Reagent Co., Ltd.	AR	98.8 wt.%	64-17-5
Ferrocene	Sinopharm Chemical Reagent Co., Ltd.	AR	0.6 wt.%	102-54-5
Thiophene	Afaisa (China) Chemical Co., Ltd.	AR	0.6 wt.%	110-02-1
Nitrogen	Jin Hong Gas Co., Ltd.	99.999%	-	7727-37-9
Cotton yarn	Shandong Jin Yue Textile Co., Ltd.	-	-	-
PET yarn	Joyful Home Textiles Co., Ltd.	-	-	-

Table S2. Yarn specifications.

Yarn type	Linear density	Twist direction/ level	Single/ply yarn specification
Cotton yarn	Approximately 59 Tex/531 Denier	Z twist/ Twist level was not provided by the supplier	20S/2, two-ply yarn
PET yarn	Approximately 29.5 Tex/266 Denier	No twist direction or twist level was provided by the supplier	20S/1, single yarn

Table S3. Performance comparison of our PET/CCY blended fabric-based wearable heater with previously reported fabric-based wearable heaters.

Wearable heaters	Air permeability (mm s^{-1})	Moisture permeability (g m^{-2} day^{-1})	Voltage (V)	Temperature (°C)	Response time (s)	Flexibility	Washability	Long-term durability	Ref.
PET/CCY blended fabric	1104.09 ± 7.69	2536.67 ± 25.45	7	147	45	Yes	60 cycles (30 h)	84 days	This work
PA-PEI-rGO textile	NA	NA	3	141	NA	Yes	NA	NA	[1]
PTFE/CNT fiber woven textile	NA	NA	5	145	>500	NA	NA	NA	[2]
TPU/graphene/AgNWs @textile	NA	NA	4	104	~125	NA	12 h	NA	[3]
Cotton fabric@PANI/ AgNWs/PDMS	NA	4272	1.1	127	180	Yes	NA	NA	[4]
Cotton@PDA/CNTs/ PFOTES	NA	NA	6	>85	<175	Yes	NA	NA	[5]
Composite graphene/CNT-based fabric	331.35	NA	5	65	28	Yes	24 h	NA	[6]
MXene-coated cellulose fabric	NA	NA	6	100	16	Yes	NA	25 days	[7]
MXene@PU/PET blend fabric	295.87	1355.03	NA	49.6	1800	NA	20 cycles	10 days	[8]
PI/TPU/AgNWs membrane	2.7 ± 0.6	NA	NA	<50	NA	Yes	10 h (5 times)	NA	[9]
Sericin-MXene-coated silk fabric	315.16	NA	13	74.6	25	NA	50 min	NA	[10]
Nanofibrillated cellulose-graphene	NA	NA	NA	60	180	Yes	NA	NA	[11]
RGO/MXene@cotton fabric	NA	NA	12	66.7	<60	NA	NA	NA	[12]
CNT-nanofibrillated cellulose membrane	NA	NA	7.8	67.3	NA	Yes	NA	NA	[13]
Graphene/nanocellulose films	NA	NA	10	145	150	NA	NA	NA	[14]
Holocellulose nanofibrils/ CNTs/cellulose aerogel fiber textile	NA	NA	2.5	179.6	40	NA	NA	NA	[15]
MXene/silver composite cellulose-based fabric	NA	NA	12	128.8	30	Yes	NA	NA	[16]
Cotton/TA/Ag NPs/PDMS textile	260.7 ± 6	NA	2	118.7	<300	NA	NA	NA	[17]
Cotton/PEDOT: PSS textile	NA	NA	40	94	90	Yes	NA	NA	[18]
CNT/cellulose/CuNW/ cotton fabric	15.92	NA	1.8	70	80	NA	NA	NA	[19]
MXene-coated fabric	NA	NA	5	263	76	NA	NA	NA	[20]

MXene coated PET textile	250-300	NA	3.5	174	25	NA	100 min	NA	[21]
PANI-coated PET fabric	NA	NA	12	30.3	<80	NA	NA	NA	[22]
CNT@silk fabric	NA	NA	25	42.8	<125	Yes	NA	NA	[23]
Graphene@PET fabric	NA	NA	14	80	<70	Yes	NA	NA	[24]
Graphene@cotton fabric	NA	NA	14	~60	60	NA	NA	NA	[25]
PPy@cotton fabric	NA	NA	8	51	NA	Yes	NA	NA	[26]
PPy/MXene-decorated PET textile	~150	NA	4	79	NA	NA	NA	NA	[27]
Weft-knitted carbonized fabric	NA	NA	4	>160	NA	NA	NA	NA	[28]
CNT-PDA cotton fabrics	NA	NA	6	120	20	NA	NA	NA	[29]
PEDOT-coated cotton fabric	NA	NA	6	45	NA	NA	NA	NA	[30]
Silica nanoparticle/cellulose acetate/silver@cotton cloth	NA	$\sim 2.34 \times 10^{-2}$	0.5	34.1	NA	NA	NA	NA	[31]
Copper nanoparticles/nylon 6 woven fabric	NA	NA	2	208.8	90	Yes	50 times (25 h)	30 days	[32]
PET yarn (warp) /MXene wire (weft) blended fabric	NA	NA	30	140	NA	Yes	24 h	NA	[33]
Cotton yarn (warp)/PEDOT: PSS@MXene@cotton yarn (weft) blended fabric	<1000	NA	2.5	196.8	NA	Yes	10 times (50 min)	120 days	[34]
PU/CB/Cu fibers (weft) /PET sewing threads (warp) blended fabric	299.42	2624.96	30	38	<150	NA	NA	NA	[35]

PET: polyester; PA: polyamide; PEI: polyethyleneimine; rGO: reduced graphene oxide; PTFE: polytetrafluoroethylene; CNT: carbon nanotube; TPU: thermoplastic polyurethane; AgNWs: Ag nanowires; PANI: polyaniline; PDMS: polydimethylsiloxane; PDA: polydopamine; PFOTES: 1H,1H,2H,2H-perfluorooctyltriethoxysilan; PU: polyurethane; PI: polyimide; TA: tannic acid; Ag NPs: silver nanoparticles; PEDOT:PSS: poly(3,4-ethylenedioxythiophene)-poly-(styrenesulfonate); CuNW: copper nanowire; PPy: polypyrrole; CB: carbon black.

References

1. Xie, L.J.; Min, P.; Ye, L.; He, P.; Yin, G.; Jin, M.; Zhang, H.B.; Yu, Z.Z. Interfacial enhancement enables highly conductive reduced graphene oxide-based yarns for efficient electromagnetic interference shielding and thermal regulation. *Carbon* **2024**, *230*, 119655, doi:10.1016/j.carbon.2024.119655.
2. Chai, J.L.; Wang, G.L.; Wang, G.Z.; Shao, R.Z.; Zhao, J.C.; Zhao, G.Q.; Park, C.B. Porous and conductive fiber woven textile for multi-functional protection, personal warmth, and intelligent motion/temperature perception. *Adv. Funct. Mater.* **2025**, 2416428, doi:10.1002/adfm.202416428.
3. Sun, Z.F.; Yang, J.J.; Li, Y.H.; Chen, Z.Y.; Ren, F.; Jin, Y.L.; Zhu, G.J.; Ren, P.G. Flexible and breathable textile-based multi-functional strain sensor for compressive sensing, electromagnetic shielding, and electrical heating. *J. Alloy. Compd.* **2024**, *983*, 173867, doi:10.1016/j.jallcom.2024.173867.
4. Tang, L.T.; Lyu, B.; Gao, D.G.; Jia, Z.T.; Zhu, J.M.; Ma, J.Z. Waste cotton fabric-derived multimodal heating textile for comfortable and reliable personal thermal management. *J. Clean. Prod.* **2023**, *425*, 138992, doi:10.1016/j.jclepro.2023.138992.
5. Ma, J.H.; Zhao, Q.L.; Zhou, Y.X.; He, P.X.; Pu, H.H.; Song, B.Q.; Pan, S.X.; Wang, Y.W.; Wang, C. Hydrophobic wrapped carbon nanotubes coated cotton fabric for electrical heating and electromagnetic interference shielding. *Polym. Test.* **2021**, *100*, 107240, doi:10.1016/j.polymertesting.2021.107240.
6. Wang, F.; Guo, Y.; Fang, Y.Z.; Chu, Y.Q.; Liu, Z.F. Programmed, electrically heated carbon-modified fabrics offer high stability, breathability and human thermal management applications. *Cellulose* **2024**, *31*, 7083-7099, doi:10.1007/s10570-024-06021-5.
7. Zhao, X.; Wang, L.Y.; Tang, C.Y.; Zha, X.J.; Liu, Y.; Su, B.H.; Ke, K.; Bao, R.Y.; Yang, M.B.; Yang, W. Smart $Ti_3C_2T_x$ MXene Fabric with Fast Humidity Response and Joule Heating for Healthcare and Medical Therapy Applications. *ACS Nano* **2020**, *14*, 8793-8805, doi:10.1021/acsnano.0c03391.
8. Dong, X.X.; Cao, Y.M.; Wang, C.; Wu, B.; Zheng, M.; Xue, Y.B.; Li, W.; Han, B.; Zheng, M.; Wang, Z.S.; et al. MXene-decorated smart textiles with the desired mid-infrared emissivity for passive personal thermal management. *ACS Appl. Mater. Interfaces* **2023**, *15*, 12032-12040, doi:10.1021/acsami.2c21696.
9. Gao, Q.; Lauster, T.; Kopera, B.A.F.; Retsch, M.; Agarwal, S.; Greiner, A. Breathable and Flexible Dual-Sided Nonwovens with Adjustable Infrared Optical Performances for Smart Textile. *Adv. Funct. Mater.* **2022**, *32*, 2108808, doi:10.1002/adfm.202108808.
10. Yang, H.W.; Cheng, L.; Yang, Q.L.; Farooq, A.; Ying, L.L.; Wang, P.; Wang,

Z.Q. Multifunctional and durable thermal management coating from sericin-MXene biohybrid on silk fabric micro-etched by deep eutectic solvent. *Appl. Surf. Sci.* **2023**, *623*, 156962, doi:10.1016/j.apsusc.2023.156962.

11. Li, X.; Shao, C.; Zhuo, B.; Yang, S.; Zhu, Z.; Su, C.; Yuan, Q. The use of nanofibrillated cellulose to fabricate a homogeneous and flexible graphene-based electric heating membrane. *Int. J. Biol. Macromol.* **2019**, *139*, 1103-1116, doi:10.1016/j.ijbiomac.2019.08.081.
12. Zheng, X.H.; Nie, W.Q.; Hu, Q.L.; Wang, X.W.; Wang, Z.Q.; Zou, L.H.; Hong, X.H.; Yang, H.W.; Shen, J.K.; Li, C.L. Multifunctional RGO/$Ti_3C_2T_x$ MXene fabrics for electrochemical energy storage, electromagnetic interference shielding, electrothermal and human motion detection. *Mater. Des.* **2021**, *200*, 109442, doi:10.1016/j.matdes.2020.109442.
13. Li, X.P.; Zou, H.J.; Zhuo, B.; Shao, C.; Cao, S.A.; Zhang, B.X.; Yuan, Q.P. Steady and robust CNTs-based electric heating membrane fabricated by addition of nanocellulose and hot-press encapsulation. *Langmuir* **2021**, *37*, 5763-5775, doi:10.1021/acs.langmuir.1c00014.
14. Yang, Y.; Luo, C.L.; Chen, X.D.; Wang, M. Sustainable electromagnetic shielding graphene/nanocellulose thin films with excellent joule heating and mechanical properties via in-situ mechanical exfoliation and crosslinking with cations. *Compos. Sci. Technol.* **2023**, *233*, 109913, doi:10.1016/j.compscitech.2023.109913.
15. Chen, Y.; Zhang, C.Z.; Tao, S.M.; Chai, H.T.; Xu, D.F.; Li, X.X.; Qi, H.S. High-performance smart cellulose nanohybrid aerogel fibers as a platform toward multifunctional textiles. *Chem. Eng. J.* **2023**, *466*, 143153, doi:10.1016/j.cej.2023.143153.
16. Li, H.; Pan, Y.N.; Du, Z.Q. Self-reduction assisted MXene/silver composite Tencel cellulose-based fabric with electrothermal conversion and NIR photothermal actuation. *Cellulose* **2022**, *29*, 8427-8441, doi:10.1007/s10570-022-04766-5.
17. Guo, Z.P.; Wang, Y.L.; Huang, J.J.; Zhang, S.Y.; Zhang, R.Q.; Ye, D.Z.; Cai, G.M.; Yang, H.J.; Gu, S.J.; Xu, W.L. Multi-functional and water-resistant conductive silver nanoparticle-decorated cotton textiles with excellent joule heating performances and human motion monitoring. *Cellulose* **2021**, *28*, 7483-7495, doi:10.1007/s10570-021-03955-y.
18. Jalil, M.A.; Ahmed, A.; Hossain, M.M.; Adak, B.; Islam, M.T.; Moniruzzaman, M.; Parvez, M.S.; Shkir, M.; Mukhopadhyay, S. Synthesis of PEDOT:PSS Solution-Processed Electronic Textiles for Enhanced Joule Heating. *ACS Omega* **2022**, *7*, 12716-12723, doi:10.1021/acsomega.1c07148.

19. Guo, Z.; Sun, C.; Wang, J.; Cai, Z.; Ge, F. High-Performance Laminated Fabric with Enhanced Photothermal Conversion and Joule Heating Effect for Personal Thermal Management. *ACS Appl. Mater. Interfaces* **2021**, *13*, 8851-8862, doi:10.1021/acsami.0c23123.
20. Wang, X.; Lei, Z.; Ma, X.; He, G.; Xu, T.; Tan, J.; Wang, L.; Zhang, X.; Qu, L.; Zhang, X. A lightweight MXene-Coated nonwoven fabric with excellent flame Retardancy, EMI Shielding, and Electrothermal/Photothermal conversion for wearable heater. *Chem. Eng. J.* **2022**, *430*, 132605, doi:10.1016/j.cej.2021.132605.
21. Liu, X.; Jin, X.; Li, L.; Wang, J.; Yang, Y.; Cao, Y.; Wang, W. Air-permeable, multifunctional, dual-energy-driven MXene-decorated polymeric textile-based wearable heaters with exceptional electrothermal and photothermal conversion performance. *J. Mater. Chem. A* **2020**, *8*, 12526-12537, doi:10.1039/d0ta03048a.
22. Zhao, Z.; Zhou, J.; Xiao, H.; Liu, Y.; Lu, M. Creation of polyaniline-coated polyester fabrics with conductive, electrothermal and energy-storage properties via micro-dissolution method. *Mater. Today Commun.* **2020**, *24*, 101042, doi:10.1016/j.mtcomm.2020.101042.
23. Zhou, J.; Zhao, Z.; Hu, R.; Yang, J.; Xiao, H.; Liu, Y.; Lu, M. Multi-walled carbon nanotubes functionalized silk fabrics for mechanical sensors and heating materials. *Mater. Des.* **2020**, *191*, 108636, doi:10.1016/j.matdes.2020.108636.
24. Ge, F.; Fei, L.; Zhang, J.; Wang, C. The Electrical-Triggered High Contrast and Reversible Color-Changing Janus Fabric Based on Double Side Coating. *ACS Appl. Mater. Interfaces* **2020**, *12*, 21854-21862, doi:10.1021/acsami.0c03194.
25. Ji, X.; Liu, W.; Yin, Y.; Wang, C.; Torrisi, F. A graphene-based electro-thermochromic textile display. *J. Mater. Chem. C* **2020**, *8*, 15788-15794, doi:10.1039/d0tc03144e.
26. Wang, B.; Cheng, H.; Zhu, J.; Yuan, Y.; Wang, C. A flexible and stretchable polypyrrole/knitted cotton for electrothermal heater. *Org. Electron.* **2020**, *85*, 105819, doi:10.1016/j.orgel.2020.105819.
27. Wang, Q.W.; Zhang, H.B.; Liu, J.; Zhao, S.; Xie, X.; Liu, L.; Yang, R.; Koratkar, N.; Yu, Z.Z. Multifunctional and Water-Resistant MXene-Decorated Polyester Textiles with Outstanding Electromagnetic Interference Shielding and Joule Heating Performances. *Adv. Funct. Mater.* **2019**, *29*, 1806819, doi:10.1002/adfm.201806819.
28. Zhang, M.; Wang, C.; Liang, X.; Yin, Z.; Xia, K.; Wang, H.; Jian, M.; Zhang, Y. Weft-Knitted Fabric for a Highly Stretchable and Low-Voltage Wearable Heater. *Adv. Electron. Mater.* **2017**, *3*, 1700193, doi:10.1002/aelm.201700193.
29. Sadi, M.S.; Pan, J.; Xu, A.; Cheng, D.; Cai, G.; Wang, X. Direct dip-coating of

carbon nanotubes onto polydopamine-templated cotton fabrics for wearable applications. *Cellulose* **2019**, *26*, 7569-7579, doi:10.1007/s10570-019-02628-1.

30. Zhang, L.; Baima, M.; Andrew, T.L. Transforming Commercial Textiles and Threads into Sewable and Weavable Electric Heaters. *ACS Appl. Mater. Interfaces* **2017**, *9*, 32299-32307, doi:10.1021/acsami.7b10514.
31. Liu, Q.; Huang, J.; Zhang, J.; Hong, Y.; Wan, Y.; Wang, Q.; Gong, M.; Wu, Z.; Guo, C.F. Thermal, Waterproof, Breathable, and Antibacterial Cloth with a Nanoporous Structure. *ACS Appl. Mater. Interfaces* **2018**, *10*, 2026-2032, doi:10.1021/acsami.7b16422.
32. Du, P.; Zhang, J.; Guo, Z.; Wang, H.; Luo, Z.; Fan, Z.; Li, B.; Cai, Z.; Ge, F. A novel breathable flexible metallized fabric for wearable heating device with flame-retardant and antibacterial properties. *J. Mater. Sci. Technol.* **2022**, *122*, 200-210, doi:10.1016/j.jmst.2022.01.015.
33. Jeong, W.; Shin, H.; Nam, H.; Cho, H.; Heo, J.; Seo, J.; Han, T.H. Nature-Inspired Design of Twisted MXene Heating Wires for Robust Wearable Heating Textiles. *Adv. Funct. Mater.* **2024**, *35*, 2418824, doi:10.1002/adfm.202418824.
34. He, G.; Ning, F.; Liu, X.; Meng, Y.; Lei, Z.; Ma, X.; Tian, M.; Liu, X.; Zhang, X.; Zhang, X.; et al. High-Performance and Long-Term Stability of MXene/PEDOT:PSS-Decorated Cotton Yarn for Wearable Electronics Applications. *Adv. Fiber Mater.* **2023**, *6*, 367-386, doi:10.1007/s42765-023-00348-7.
35. Zhuang, J.; Liu, Y.; Bian, D.; He, F.; Li, Y.; Fan, W. Spinning solution viscosity reducing and wet spinning of carbon black-based elastic conductive fibers for sports monitoring and healthcare electrical heating. *J. Mater. Res. Technol.* **2025**, *36*, 1-12, doi:10.1016/j.jmrt.2025.03.096.
